# Gut Microbiome Dynamics in Food Allergy Development Across the Lifespan: Microbial Mechanisms, Host Interactions, and Therapeutic Perspectives

**DOI:** 10.3390/microorganisms14050970

**Published:** 2026-04-25

**Authors:** Aaron Wilson, Brian Quach, Khalia Musa, Ibrahim Musa

**Affiliations:** 1 Department of Pathology, Microbiology & Immunology, New York Medical College, Valhalla, NY 10595, USA; awilson24@student.touro.edu (A.W.); 2 School of Medicine, Quinnipiac University, Hamden, CT 06518, USA

**Keywords:** gut microbiome, inflammatory diseases, lifespan development, immunology, bacterial functions, food allergy

## Abstract

Over the past several decades, the gut microbiome (GM) has been the focus of extensive investigation. In recent years, major discoveries such as the role of maternal breastfeeding in infant GM development and mode of delivery on infant GM health have expanded scientific knowledge on this topic. As this is a rapidly expanding field of research, substantial work remains to further elucidate and integrate the existing evidence on its role in allergic response and immunological development. This comprehensive review will examine the latest discoveries in GM research and its role in the development of food allergies across the lifespan. Examining the existing literature may identify knowledge gaps regarding precise mechanisms through which the development of GM influences the maturation of the immune system. Given the abundance of the literature, we conducted a database search for articles published within the past 10 years. A total of 56 original research articles were retrieved, analyzed, and included in our review. This review article aims to integrate the current evidence on understanding how the development of GM impacts the immune system and food allergy response throughout the lifespan. We aim to uncover microbial mechanisms of allergy response, host and microbe interactions, and opportunities for therapeutic intervention. Additionally, we aim to reveal gaps in the current knowledge of the GM’s influence on allergy development, offering directions for future research.

## 1. Introduction

Between 1997 and 2011, there was a significant increase in the prevalence of food allergies, rising by up to 50 percent [1]. As of 2023, the Centers for Disease Control and Prevention reported that more than one in four children in the United States suffer from seasonal allergies, eczema, or food allergies. Over the past few decades, extensive research has focused on the human gut microbiome (GM) and its impact on our health [2]. It is now evident that the development of the GM starts during pregnancy with maternal nourishment and continues to evolve throughout life, influenced by environmental factors and diet. These factors play a crucial role in immune development and response, potentially leading to allergies, or allergy-based illnesses such as asthma or atopic dermatitis.

Several studies have shown a connection between gut bacteria and food allergies in infants and children, including the study by Zongxin et al., which showed that certain types of bacteria in infant stool were linked to allergies caused by IgE antibodies [3]. The study found that higher levels of *Clostridium sensu stricto* and *Anaerobacter* and lower levels of *Bacteroides* and *Clostridium XVIII* could disrupt the immune balance in infants and produce IgE antibodies against food proteins [3]. Another study by Chien-Chang Chen showed that children with food allergies had a lower diversity of gut bacteria than healthy children [4]. These children showed a significant decrease in the number of Bacteroidetes bacteria and a significant increase in the number of *Bacillota* compared to healthy children [4]. At the genus level, there were significant increases in the numbers of *Sphingomonas*, *Sutterella*, *Bifidobacterium*, *Collinsella*, *Clostridium sensu stricto*, *Clostridium IV*, *Enterococcus*, *Lactobacillus*, *Roseburia*, *Faecalibacterium*, *Ruminococcus*, *Subdoligranulum*, and *Akkermansia* in the group of children with food allergies [4]. There were also significant decreases in the numbers of *Bacteroidetes*, *Parabacteroides*, *Prevotella*, *Alistipes*, *Streptococcus*, and *Veillonella* in this group. Additionally, the analysis revealed that certain types of bacteria (specifically increased abundances of *Clostridium IV* and *Subdoligranulum*, and decreased abundances of *Bacteroidetes* and *Veillonella*) could potentially be used to identify food allergies [4]. Azad et al. investigated the connection between gut bacteria and food sensitization in 1-year-old infants [5]. They assessed food sensitization through skin prick testing on 166 infants and collected fecal samples at 3 and 12 months. The study found that infants with food sensitization had an overrepresentation of *Enterobacteriaceae* and an underrepresentation of *Bacteroidaceae* in their gut microbiota at 3 months and one year [5]. Additionally, lower microbiota richness was observed at 3 months [5]. This suggests that early gut colonization might contribute to the development of atopic diseases, including food allergies. Additionally, if a mother takes antibiotics after giving birth and continues breastfeeding, small amounts of the antibiotics can pass into her breast milk, potentially altering the baby’s developing GM. Maternal antibiotic prophylaxis can influence neonate microbiomes through the umbilical cord or during birth [6,7]. Together, these factors demonstrate how antibiotics can affect both the developing microbiome of the child and the mother, highlighting the delicate interaction between maternal and neonatal microbiomes.

There has been a sharp increase in allergic disease over the last four decades, particularly in developed countries. During this time, there have been marked improvements in sanitation, an increase in the use of antibiotics, and changes in diet in these developed countries, which have been linked to the incidence of allergic diseases. About 10–100 trillion microbes and 1000 bacterial species exist in the human gut. Alterations in the commensal bacteria can cause changes to the immune system, resulting in food allergies. There is evidence indicating that there are differences in GM composition between patients with non-food allergies and those with IgE-mediated food allergies. One study involving 233 allergic patients under the age of four found that the microbiomes of patients with IgE-mediated food allergies included *Collinsella aerofaciens*, *Dorea formicigenerans*, unclassified *Methanobrevibacter*, *Blautia obeum*, and *Coprococcus catus* [8]. In contrast, the non-allergic children in the same study exhibited a more diverse microbiome, comprising 18 different species, with *Bifidobacterium adolescentis* being the most prevalent [8]. However, it is unclear how dysbiosis, the change in microbiota composition that leads to disruption of gut homeostasis and eventual disease, affects the immune system in the development of food allergies. Our review of the existing literature highlights a significant gap in understanding the precise mechanisms by which microbiota development influences immune system maturation in infants and its long-term implications for adulthood. This review aims to provide a comprehensive review of the current knowledge on the mechanisms by which changes in the gut microbiota in utero through childhood and adulthood lead to alterations in the immune system, causing food allergies.

## 2. Methods

In this review, we searched the NIH PubMed database and the New York Medical College database using keywords such as “gut microbiome”, “food allergies”, “inflammatory disease”, “clinical trials”, “probiotics”, and “mode of delivery.” Articles published within the last 10 years were used to demonstrate the evolution of research on the gut microbiome and our understanding of food allergies. Articles published more than 10 years ago were excluded from analysis. A total of 90 articles were reviewed. We screened for human models used in examining food allergies and the environments in which these allergies manifested. Moreover, we included a screening for the environmental elements that may have facilitated allergy production. Of these 90 articles, a total of 51 original research articles were retrieved, analyzed, and included in our review. Moreover, we included comprehensive analyses, systematic reviews, and reviews to enhance our understanding of how the gut microbiome develops and changes throughout life. Although there were a multitude of murine model studies that examined the gut microbiome and food allergies, our goal was to focus on the human gut microbiome and how food allergies manifest under the circumstances outlined in this review.

## 3. Findings

### 3.1. Onset of Allergies in Newborns Is Linked to the Diversity of Their Gut Microbiome

**Figure 1:**
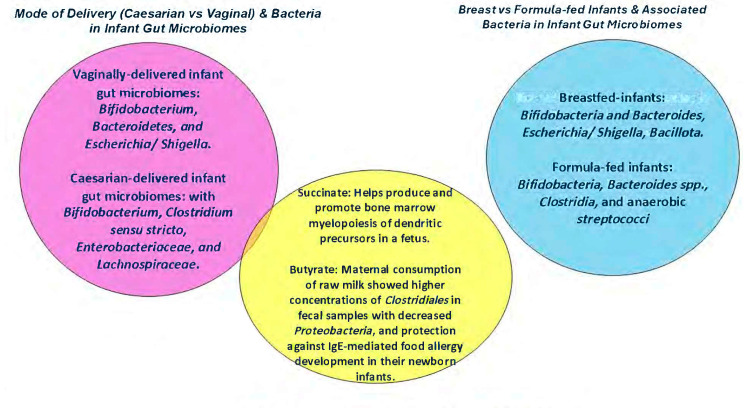
Bacteria in infant gut microbiomes are associated with the mode of delivery (cesarean vs. vaginal), as well as with breast-fed vs. formula-fed infants, and short-chain fatty acids. Original image created with www.biorender.com.

A newborn child acquires approximately half of its GM and immunity from the mother. In the “Enquiring About Tolerance (EAT)” study, Marrs aimed to investigate whether regularly consuming allergenic solid foods could reduce the prevalence of allergies compared to breast-fed children. In their randomized control trial from 3 months of age, a total of 1303 exclusively breast-fed infants were enrolled in the trial. Samples were collected at baseline, and additional ones were sampled and selected along with controls at 6 and 12 months of age to examine the changes in their gut microbiomes using 16S ribosomal RNA gene-targeted amplicon sequencing. For the group to evaluate how the infant gut microbiome was assembled, 288 patients provided baseline samples, 218 provided only baseline samples, and a subset of 70 provided samples from time points 3, 6, and 12 months. Subset groups were cases and controls, which included 42 participants as a standard introduction group and 28 as an early introductory group. A total of 28 participants consumed six allergenic foods (boiled hen’s egg, peanut, cow’s milk (yogurt), wheat, white fish, and sesame) with food-specific IgE levels measured during the study. This was compared to another group consisting of 42 participants that was exclusively breast-fed. The infants were examined for atopic dermatitis at their enrollment visit at 3 months and 12 months old, using a UK diagnostic criteria-based photographic protocol of the International Study of Asthma and Allergies in Childhood Phase Two. The results showed that 12 patients developed an egg allergy, one had a milk allergy, and one had a codfish allergy, with 13 developing atopic dermatitis. Upon examining the microbial communities of the three-month-old infants, Marrs et al. found that *Bifidobacterium*, *Bacteroidetes*, and genera of *Bacillota* and *Pseudomonadota* were present. The exclusively breast-fed infants were reported to have the most diverse microbiomes, containing Bifidobacterium-rich and *Bacteroidetes*-rich clusters and *Escherichia*/*Shigella*, with genera featuring *Bacillota*. Marrs showed that the microbiome of infants delivered vaginally predominantly consisted of *Bifidobacterium*, *Bacteroidetes*, and *Escherichia*/*Shigella*, compared to cesarean-born infants with *Bifidobacterium*, *Clostridium sensu stricto*, *Enterobacteriaceae*, and *Lachnospiraceae bacterium*. The amount of *Bacteroidetes* found in cesarean-delivered infants was significantly less than that of those delivered vaginally. *Bacteroides fragilis* has polysaccharide A molecular moieties that allow for the development of regulatory T cells in the lamina propria [9]. Research on GM composition reveals that babies delivered via cesarean often have a higher presence of pathogenic bacteria in their intestines. These bacteria, which can be acquired from healthcare environments, include *Enterococcus*, *Enterobacter*, and *Klebsiella* [10]. The presence of these pathogens can disrupt the delicate balance of the Th1/Th2 immune response, potentially increasing the risk of developing allergies during the crucial first 1000 days of life. In contrast, infants born vaginally tend to develop a GM that is more similar to their mother’s vaginal microbial community [11]. See Figure 1 for a summary on the effects that the mode of delivery and breast-fed versus formula-fed infants have on the development of food allergies in infants. For example, if a mother’s vaginal microbiome contains *Lactobacillus* species, her infant is more likely to acquire some bacteria during birth [8]. This early microbial transfer may contribute to a lower risk of allergy development in the newborn due to probiotic effects of the commensal bacteria. See Table 1 for a summary of key influencing factors, microbial signatures, immune changes, and associated allergies in newborns.

### 3.2. Gut Microbiome and Development of Allergies in Adolescence

During puberty, the changes in sexual maturation result in fluctuations in testosterone and estrogen levels, which play a crucial role in shaping the GM. Studies have shown that these hormonal changes lead to alterations in the composition and diversity of the GM [12,13]. Studies comparing teenagers following a Westernized diet and a Mediterranean diet have revealed marked differences in their gut microbiota. Teenagers on a Westernized diet exhibited higher levels of compounds associated with amino acid and lipid metabolism, whereas those on a Mediterranean diet had higher levels of short-chain fatty acids, indicating differing functional outcomes based on diet [14]. Furthermore, stress has been found to reduce the levels of beneficial bacteria such as *Lactobacillus* and *Bifidobacterium*, while increasing the presence of Gram-negative bacteria. The latter produce lipopolysaccharides and activate toll-like receptors, leading to inflammatory responses that can damage gut integrity and disrupt the production of short-chain fatty acids such as butyrate and acetate [15]. Collectively, these factors contribute to gut dysbiosis and could potentially lead to the development or exacerbation of allergies.

In adolescents who have a high intake of processed foods, high sugar, or foods high in fat, it is linked to obesity, along with being in a chronically inflammatory state. In turn, cytokine production is increased and can impact daily mental health [16]. This is likely due to the amount of sugar an average American adolescent consumes, which is 41 g per day, and eight times the recommended amount for daily consumption [17]. This also increases the risk of diabetes onset in adolescents and may add more psychological stress to their development. In early-life rats, it was shown that high consumption of sugar alters their gut microbiome, which was independent of body weight, adiposity, and total caloric intake across fructose-to-glucose ratios [18]. Adolescents may also engage in alcohol consumption, which can disrupt normal gut microbiome development and contribute to worsening mental health, notwithstanding an already inflamed system. Neurotransmitters, which include serotonin, gamma-aminobutyric acid (GABA), dopamine, and norepinephrine, also play either a direct or indirect role in the brain–gut microbiota axis in adolescents, which can impact behavioral responses [19] and be modulated at the mercy of metabolites, which, when depleted, make major depressive disorder onset more likely [20].

Despite the occurrence of stress and other bodily disturbances, there have been limited studies investigating the interaction between biological processes such as hormone changes during adolescence, social influence, and mental health, and their impact on GM development. For example, Fiorentino et al. demonstrated that a brain with autism spectrum disorder (ASD) had altered gene expressions in the blood–brain barrier (BBB), neuroinflammation, and implications of changes in the gut microbiome [21]. In adolescence, exposure to westernized diets and alcohol may trigger mental health conditions, which may subsequently have adverse outcomes on GM development. Greater emphasis in future research may focus on examining how biological, dietary, and psychosocial factors influence the development of allergies during adolescence. See Table 1 for a summary of key influencing factors, microbial signatures, immune changes, and associated allergies in adolescence.

### 3.3. Gut Microbiome and Development of Allergies in Adults

In adulthood, a healthy GM is influenced by foods, whether the diet is animal- or plant-based. Plant starches rich in amylopectin or amylose can be metabolized by species such as *Bifidobacterium*, *Bacteroidetes*, and *Fusobacteriota* spp. [22]. However, it should also be mentioned that people who consume plant-rich carbohydrate-based diets tend to have more *Prevotella* bacteria in their gut, while those consuming more animal fat and proteins have higher *Bacteroidetes* levels. The recent literature has reported that as humans age, important gut bacteria such as *Faecalibarterium prausnitzii* decrease, while other more pathogenic bacteria including *E. coli*, *Pseudomonadota*, and *Staphylococcus* increase [22]. Furthermore, there is an increased risk of DNA damage, stress response, and a compromised immune system, marking a more pro-inflammatory GI microbiome [23,24]. These preceding studies offer an understanding of a potential mechanism of the GM changes from early adulthood to late adulthood. The composition of the GM has been linked to the development of food allergies later in life, but there are limited studies on the specific factors contributing to this connection. A prior study has examined the aspects of culture, diet, and aging of the adult microbiome [25]. Hamdi et al. [25] examined the dietary effects of the gut microbiota in an elderly population in rural and urban Vietnam. The findings revealed that age-related reductions in *Roseburia*, *Veillonella*, and *Pseudomonadota* were observed in elderly populations, see Table 1. Rural participants showed a reduction in butyrate-producing genera, suggesting a link to traditional foods in the region, and urban participants demonstrated a great abundance of health-associated genera, indicating plant-based diets. Newer studies should consider examining the roles of these factors together to see if they are correlated to speed or slow the decline of the adult microbiome and initiate allergy development in different populations. See Table 1 for a summary of key influencing factors, microbial signatures, immune changes, and associated allergies in adults.

**Table 1:** Life stage-specific gut microbiome alterations and associated allergic outcomes.

Life Stage	Key Influencing Factors	Microbial Signatures (↑/↓)	Functional/Immunological Effects	Associated Allergic Outcomes	Representative Evidence
Prenatal/Perinatal	Maternal diet, antibiotics, delivery mode, breastfeeding	↑ *Enterococcus*, *Enterobacter* (C-section); ↓ *Bacteroidetes*; ↑ *Bifidobacterium* (vaginal)	Treg induction; immune priming; Th1/Th2 balance disruption	Food allergy, atopic dermatitis	Marrs et al.; Azad et al.
Infancy (0–2 years)	Breastfeeding, antibiotics, early diet	↑ *Enterobacteriaceae*; ↓ *Bacteroidaceae*; ↓ diversity	Impaired tolerance; ↑ IgE; altered SCFAs	Food sensitization, eczema	Zongxin et al.; Chen et al.; Azad et al.
Childhood	Diet, environment	↑ *Bacillota*; ↓ *Bacteroidetes*; altered genera	Immune dysregulation; ↓ SCFAs	Persistent food allergy, dermatitis	Chen et al.
Adolescence	Hormones, diet, stress, alcohol	↓ *Lactobacillus*, *Bifidobacterium*; ↑ Gram-negative bacteria	Inflammation; TLR activation; gut–brain axis	Allergy exacerbation	Fiorentino et al.
Adulthood	Diet, aging, obesity	↓ *Faecalibacterium*; ↑ *Pseudomonadota*; ↑ *Bacillota*/*Bacteroidetes*	Pro-inflammatory state; ↓ butyrate	Adult-onset allergy	American Gut Project
Elderly	Aging, diet	↓ *Roseburia*, *Prevotella*, *Veillonella*	Immune senescence; ↓ SCFAs	Inflammatory/allergic disease	Hamdi et al.

**Figure 2:**
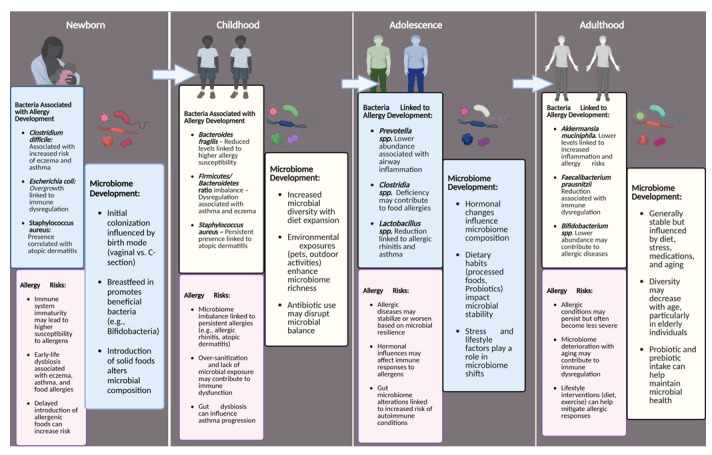
Illustration of microbiome evolution and allergy risks across life stages: newborn, childhood, adolescence, and adulthood.

Publicly available resources such as the American Gut Project questionnaire and fecal rRNA sequence data were analyzed to identify the relationship between dysbiosis and allergy development in American adults [26]. Findings from this analysis revealed that adults with low diversity, reduced *Clostridiales* spp., and increased *Bacteroidetes* ssp. in gut microbiota may be suggestive of an acquired nut or seasonal allergy. Another study found that adults with obesity showed differing biota than their average weight counterparts. Obese adults were found to have elevated counts of *Bacillota*, *Fusobacteriota*, *Pseudomonadota*, and *Lactobacillus*, in addition to an elevated *Bacillota*/*Bacteroidetes* ratio displayed similarly in animal studies, while also having less *Akkermansia muciniphila* and *Faecalibacterium prausnitzii* [27]. These findings may inform therapies utilizing gut microbiota modulation for treating obesity. Figure 2 summarizes the evolution of the gut microbiome from infancy to adulthood and the various changes in composition that occur. These studies emphasize the significance of bacterial diversity in the human gut microbiome. They suggest that people living in urban, suburban, and rural areas may have varying risks of developing allergies based on their location and the types of bacteria present in their microbiomes. Obesity, maintaining a healthy weight, and a diet containing important short-chain fatty acids such as butyrate can also aid in supporting the development of a healthy gut microbiome and reducing the likelihood of allergic disease development in humans.

This Figure 1 summarizes the effects that the mode of delivery and breast-fed versus formula-fed infants have on the development of food allergies in infants. The findings across these studies show that GM development is shaped as early as the neonatal period and during the birthing process. Depending on the mode of delivery, methods such as cesarean section may introduce new bacterial species through increased maternal skin contact. As early exposure to some species has been identified as helpful for the baby’s health, others may be pathogenic and could contribute to the development of allergies later in life. Future studies may benefit from isolating specific species to better define the precise role of individual organisms.

### 3.4. Interventions, Modifying Factors of the Gut Microbiome, and Their Effects on Allergy Development

#### 3.4.1. Probiotics and Prebiotics in Maintaining Gut Health and Their Impact on Allergy Prevention

Probiotics and prebiotics play a crucial role in maintaining gut health at all stages of life. Probiotics are beneficial microorganisms, and prebiotics are compounds that promote their growth and support gut health in various ways [28]. During pregnancy, probiotics can prevent gestational diabetes, postpartum depression, and mastitis. They also improve the gut health of newborns by altering the vaginal and breast milk microbiome [29]. Furthermore, they enhance neuropsychological performance in children, alleviate atopic dermatitis, and reduce inflammation and cognitive decline in the elderly [30]. Additionally, probiotics help regulate immune responses, decrease IgE levels and increase IgA, which are important for allergy prevention [30]. The use of prebiotics and probiotics in treating food allergies remains a subject of controversy, with conflicting evidence regarding their effectiveness, dosage, method of administration, and duration of treatment. A systematic review by Santos et al. concluded that experimental studies indicate probiotic use may promote immunomodulation and alleviate clinical symptoms. However, there is still a need for clarity on the appropriate dosage, timing, and administration route to ensure efficacy [31]. Notably, the bacteria that appeared to provide the most significant benefits were *Bifidobacterium breve* and *B. lactis* [31]. Additionally, two studies referenced in the review revealed that children given *Lactobacillus rhamnosus* showed decreased IgE levels in response to allergies to cow’s milk and peanuts [31]. The authors also mentioned a study in which a group received casein along with an LCG supplement for six months, resulting in increased butyrate levels [32]. Despite the promising results from these studies, they did not specify the dosages administered to the children or the frequency of administration. These factors may be crucial in effectively modulating the GM and treating food allergies in children, see Table 2. Therefore, further studies are needed to confirm the efficacy of these interventions.

#### 3.4.2. Emerging Interventions for Allergy Treatment

Microbiome-based therapies are an exciting frontier in treating allergic diseases. They focus on utilizing the GM to regulate the immune system. Current strategies include using probiotics and prebiotics to encourage the growth of beneficial microbial communities and fecal microbiota transplantation (FMT) to restore microbial balance in allergic individuals [33]. A study by Huang et al. found that FMT in BALB/c mice with an allergy to OVA was able to alleviate the condition compared to the control group that was not given FMT [34]. Human clinical trials are ongoing; however, they remain only in phase I with the aim of assessing the tolerability of oral FMT in patients with peanut allergies in patients aged 18–40 (NCT02960074). Postbiotics, particularly short-chain fatty acids, are being studied for their potential to reduce inflammation and improve immune tolerance. Synbiotics and genetically engineered bacteria designed to deliver specific therapeutic molecules are also being explored. Dietary changes that affect microbiome composition, alongside the development of microbiome-derived metabolites and targeted bacteriophages, provide further opportunities for intervention [35]. These approaches demonstrate the potential for personalized microbiome-based therapies to transform allergy management by targeting the underlying microbial–immune interactions. Such interactions may include induction of T-regulatory cells, SCFA production, and pattern recognition receptor signaling. As previously stated, diet plays a significant role in shaping the gut microbiome. Plant-based and fiber-rich diets may allow for a more diverse microbiome and lower the risk of inflammation throughout the body and allergy onset. In comparison to diets that contain preservatives, additives, or are high in fat, these may favor the development of a microbiome that promotes inflammation, dysbiosis, or exacerbates food allergies. Future studies should conduct direct comparisons and or contrasts that examine the populations of bacteria that occupy patient gut microbiomes that adhere to these diets and confirm the outcomes associated with diets that contain preservatives, additives, or high-fat.

#### 3.4.3. The Indoor Environment and Its Influence on the Gut Microbiome

With the growing emphasis on humans spending more time indoors, the microbiome may change to represent this. In a study conducted by Fu et al., they assessed the dormitories of 86 rooms at Shanxi University. They concluded that *Sphingomonas*, *Caulobacter*, uncharacterized *Caulobacteraceae* and *Comamonadaceae* were associated with rhinitis in dormitories [36]. The group also found that higher levels were associated with higher indoor particulate matter (PM_2.5_) concentrations and were associated with more bacterial taxa that were protective from rhinitis than those that lived on the lower levels, demonstrating a lower level of bacterial taxa that exhibited elevated risk of rhinitis [36]. This study also showed that indoor curtains and higher CO_2_ concentrations exhibited lower levels of rhinitis-promoting bacteria [36]. This study was able to introduce a novel way of understanding how complex environmental factors can influence the indoor microbiome and innovate strategies to control it.

#### 3.4.4. Fungi and Their Influence on Allergic Disease Development

In addition to bacterial species, other microorganisms such as fungi are associated with the GM and allergy development. Such fungi include the *Candida*, *Rodotorula*, *Issatchenkia*, *Malassezia*, and *Sarccharomyces* genera [37]. It is noted that species such as *Issatchenkia* and *Rhodotorula* are of higher abundance in adolescent allergy development; however, in adulthood, the most prevalent fungal contributor is *Candida albicans*. As *Candida* species are typically referred to as opportunistic pathogens, they are known to induce Th17, modulating regional immune responses. Of these fungal genuses, *Malassezia* predominates on the skin and uses the skin lipids for nutrients [38]; this would normally secrete antimicrobial products to prevent pathogenic bacterial growth [39]. However, in the context of *Malassezia furfur*, it has been implicated in atopic dermatitis (AD) onset in Head and Neck Dermatitis (HND), which is a refractory phenotype of AD [40]. Chu et al. found that an increased sensitization to *M. furfur* was associated with HND onset and increased disease severity and may trigger the release of inflammatory cytokines [40].

#### 3.4.5. The Virome and Its Influence on Allergic Disease Development

The role of viruses within the GM and allergy development remains incompletely understood, with ongoing research aimed at improving understanding. One major contributor to the viral division of the GM is eukaryotic viruses. Such species include *Enterovirus*, *Rotavirus*, *Norovirus*, *Astrovirus*, *Bocavirus*, *Cyclovirus*, and *Picobirnavirus*, among others [41]. Though many viral species are recognized as causes of pathogenic infection, these viruses may play a beneficial role in gut homeostasis through mechanisms such as anti-inflammatory cytokines [41]. Moreover, the gut virome in infants can be different compared to that of others. A study conducted by Perdue et al. examined the gut virome of 131 infants from high-atopy-risk suburban and urban environments and compared them against family farming communities. Their study found that while the viromes were similar at 12 months old, they differed significantly at six months of age [41]. *Mastadenovirus* and *Bocaparvovirus* were found in the stools of urban infants at 6 months of age and increased the likelihood of atopic disease onset, while *Bifidobacterium longum* subsp. *infantis* (*B. infantis*), a protective bacteriophage from atopic onset, was more prevalent in the stools of the Old Order Mennonite (OOM) Amish community infants [42]. While fungi have a dynamic interplay with the skin, especially in using the skin lipids for nutrients, future studies should consider examining what mechanisms, if any, can cause a shift from being beneficial to detrimental in the context of allergy development or even atopic dermatitis.

#### 3.4.6. Major Research Gaps

Despite increasing research on probiotics and prebiotics, there are still critical gaps that remain. The effects of different strains and underlying mechanisms are not yet fully understood. Additionally, most studies are short-term, leaving questions about the long-term safety and efficacy of probiotics and prebiotics, especially in vulnerable populations. It is important to note that individual responses to probiotics and prebiotics can vary based on genetics, diet, and existing microbiota, highlighting the need for personalized approaches. While these supplements may influence gut microbiota, we still need further study to fully understand their precise effects on microbial diversity and function. Moreover, the interactions of probiotics and prebiotics with medications and other supplements are not well explored. To establish clear guidelines for their use, we need more high-quality clinical trials, and standardized production and labeling practices are essential to ensure product consistency. Finally, the potential benefits of probiotics and prebiotics beyond gut health, such as their effects on the skin and respiratory tract, are not fully explored [43]. One probiotic that shows promise is *Lactobacillus rhamnosus* GG, which was demonstrated to aid in microbiome diversity, repair the intestinal lining, and reduce inflammation in patients with short bowel syndrome (SBS) [20]. *Bifidiobacterium breve* M-16V is another promising avenue for allergy relief, as it was shown to alleviate anaphylactic burden in mice that received transplanted fecal matter from three children with cow’s milk allergy [44].

An additional area that may warrant more exploration for future studies includes the effects of prebiotics and probiotics on allergy prevention and immunologic maturity [45,46]. As reflected in the current literature, there are few studies that have examined the effects of these supplements on environmental food allergies and hypersensitive reactions such as eczema. Despite the substantial number of clinical trials reported in the literature, heterogeneity in microbial strains across studies remains a significant point of discussion [47]. Challenges that may contribute to the reported heterogeneity may include lack of control for supplementation intervals, baseline gut microbiota composition, host genetic composition and physiology, and microbial characteristics such as chemical structure and strain effectiveness. Future investigations should prioritize controlling for these variables to generate more reliable and robust evidence than is currently available in the literature. While the exact role of viruses in the gut microbiome remains insufficiently comprehended, the studies that have examined infant urban and suburban viromes have examined how their exposures to certain environments can offer protection from allergy development. These studies offer valuable insight into what bacteriophages and viruses occupy their microbiomes. Furthermore, they articulated that their viromes changed between 6 and 12 months of age between the studies mentioned. Future studies should examine what initiates these changes within their environments, outside the introduction of solid foods, and if there are other specifics that directly or indirectly affect the virome and subsequently allergy development.

## 4. Discussion

There are still many opportunities to explore how the GM and its development influence the immune system’s strength and allergy onset. While earlier sections of this review describe associations between microbial composition and allergic phenotypes, a deeper mechanistic understanding is required to explain causality. Recent advances highlight that gut microbiome-derived metabolites, host–microbe signaling pathways, and epigenetic regulation collectively govern immune maturation and allergic sensitization.

Healthy gut bacteria, diet, and culture all play an integral role in the development of the GM, as many studies have outlined the clinical markers associated with these factors. A central mechanistic pathway linking microbiome composition to immune function involves short-chain fatty acids (SCFAs), including acetate, propionate, and butyrate, which are produced through microbial fermentation of dietary fiber. These metabolites act as key immunomodulators, promoting the expansion of colonic FoxP3^+^ regulatory T cells (Tregs) and enhancing immune tolerance to dietary antigens [48]. The future endeavors of understanding and treating the GM of young children must start with pregnant mothers by equipping them with early interventions such as access to proper prenatal care and diet. Importantly, SCFAs also influence T-helper cell differentiation. Experimental models demonstrate that adequate SCFA levels support balanced Th1/Th17 responses, whereas reduced SCFA production contributes to a Th2-skewed immune phenotype, a hallmark of allergic disease [49]. This provides a mechanistic explanation for clinical observations linking reduced abundance of butyrate-producing bacteria such as *Faecalibacterium prausnitzii* to increased allergy susceptibility.

As people age, their GMs start to change, leading to the need to investigate associated factors such as diet, lifestyle, and environment. In parallel, microbial interactions with the host immune system are mediated through pattern recognition receptors (PRRs), particularly Toll-like receptors (TLRs). Microbial-associated molecular patterns such as lipopolysaccharides (LPS) activate TLR signaling pathways, regulating cytokine production and maintaining epithelial barrier integrity [50]. However, dysbiosis—characterized by expansion of *Pseudomonata* can lead to excessive PRR activation, promoting chronic inflammation, barrier dysfunction, and increased antigen penetration, thereby facilitating allergic sensitization [51]. They showed that children with food allergies had different GMs compared to those without food allergies [51]. This highlights the importance of controlled microbial signaling during early immune education, particularly in the first 1000 days of life. Disruptions such as antibiotic exposure, cesarean delivery, and reduced microbial diversity may impair Treg induction and promote persistent Th2 polarization. This aligns with previous evidence indicating that children of mothers who took antibiotics during or outside of pregnancy were more likely to develop food allergies or sensitization [51]. Aitoro’s review also highlighted the modifiable environmental factors in using the GM in food allergies [45]. They suggested that the “first 1000 days” of life, from intrauterine development to the first two years, as well as maternal diet and breastfeeding, have a direct and indirect impact on infant microbiome development. This, along with exposure to rural environments, fiber, and fermented foods, is thought to have a protective role against allergy development. Their conclusion is that the environment during this time plays a crucial role in determining the infant’s immune system development. In essence, the environment a mother and her in utero and subsequent newborn child are exposed to is crucial.

There are other pathways within the GM and pro-inflammatory routes that also need to be explored. Beyond signaling pathways, emerging evidence underscores the role of epigenetic regulation in mediating microbiome–immune interactions. SCFAs, particularly butyrate, function as histone deacetylase (HDAC) inhibitors, altering chromatin accessibility and gene transcription. This epigenetic modulation enhances expression of genes involved in immune tolerance while suppressing pro-inflammatory and pro-allergic pathways [52,53]. These microbes included *Ruminococcus gnavus* and *Faecalibacterium prausnitzii* of the *Bacillota* phylum, but there was a reduction in *Bifidobacterium*, specifically *B. longum*, which contributed to the development of allergies [53]. Such epigenetic mechanisms are particularly critical during early life, when immune programming is highly plastic, suggesting that early microbiome perturbations may have long-term consequences on allergic disease risk. See Table 3 for a list of key microbial taxa associated with the risk of allergy onset versus microbial taxa that exhibit protective effects. SCFAs play a crucial role in regulating the immune response through various cell types [54]. Research has shown that SCFAs are linked to the expansion of colonic FoxP3+ T cells. Collectively, these findings demonstrate that the balance between Th1, Th2, and regulatory immune responses is not solely determined by microbial composition, but by the functional output of the microbiome and its downstream signaling pathways. More specifically, they discovered that mice fed these SCFAs exhibited a selective differentiation of T cells into Th1 or Th17 effector T cells [54]. Across the lifespan, these mechanisms remain dynamic and are influenced by diet, hormonal changes, environmental exposures, and aging. For example, Westernized diets low in fiber reduce SCFA production, whereas high-fiber or Mediterranean diets enhance microbial diversity and anti-inflammatory signaling.

Consequently, a deficiency in these SCFAs in the diet may lead to a shift toward a Th2-dominant response, which could increase the likelihood of developing allergies. Despite these advances, most current studies remain observational, and there is a critical need for longitudinal and interventional studies to establish causality and define precise molecular pathways. Additionally, inter-individual variability in microbiome composition and host genetics presents challenges for translation into universal therapies. From a clinical perspective, these mechanistic insights support the development of targeted microbiome-based interventions, including SCFA supplementation, precision probiotics, and engineered microbial therapies. Such approaches aim not only to restore microbial composition but also to modulate immune signaling pathways directly. In conclusion, the gut microbiome exerts a profound influence on allergic disease through integrated mechanisms involving SCFA signaling, PRR activation, immune polarization, and epigenetic regulation. A mechanistic understanding of these pathways will be essential for advancing preventive and therapeutic strategies in food allergy and related atopic diseases.

**Table 3:** Key microbial taxa associated with allergy risk versus protection.

Category	Microbial Taxa	Functional Role	Immunological Impact	Association with Allergy
Protective	*Bifidobacterium* spp.	SCFA production; gut barrier support	↑ Treg cells; ↓ IgE	Reduced food allergy risk
Protective	*Faecalibacterium prausnitzii*	Butyrate production	Anti-inflammatory; epithelial integrity	Protective against inflammation/allergy
Protective	*Bacteroidetes fragilis*	Polysaccharide A production	Treg induction; immune tolerance	Reduced allergy susceptibility
Protective	*Lactobacillus* spp.	Immune modulation; lactic acid production	↓ IgE; ↑ IgA	Improved allergy outcomes
Risk-associated	*Psuedomonadota* (e.g., *E. coli*)	LPS production	↑ Inflammation; TLR activation	Increased allergy risk
Risk-associated	*Clostridium sensu stricto*	Dysbiosis-associated metabolism	Immune imbalance	Linked to IgE-mediated allergy
Risk-associated	*Enterobacteriaceae*	Opportunistic expansion	Reduced microbial diversity	Associated with sensitization
Risk-associated	*Candida albicans*	Opportunistic fungal pathogen	Th17 activation	Associated with allergic inflammation

## 5. Future Directions and Challenges

### 5.1. Knowledge Gaps

We have revealed significant gaps in our understanding of the microbiome’s impact on different life stages, particularly in relation to the development of allergies. Further studies are imperative to elucidate the ways in which prenatal factors, early-life nutrition, and the use of antibiotics contribute to shaping the microbiome and affecting the risk of developing allergies. Moreover, there is a pressing need for more comprehensive insights into the progression of the microbiome from infancy to adulthood and its implications for immune tolerance and allergic reactions. Although the studies referenced in our review indicate potential associations, it is crucial to point out that they do not determine whether an altered GM causes food allergies. Therefore, there is a pressing need to initiate clinical or interventional trials to confirm whether the presence or absence of specific bacteria in the GM is responsible for food allergies.

### 5.2. Clinical Applications

Microbiome-based therapies are showing significant potential for advancing personalized medicine, especially in the context of managing allergies. In this regard, probiotics, prebiotics, and fecal microbiota transplantation are currently under investigation as potential therapeutic options. The transition from research to clinical implementation, however, entails the need for comprehensive trials to establish the safety and effectiveness of these interventions. Furthermore, the development of tailored treatments based on individual microbiome profiles will be essential for optimizing outcomes in personalized medicine. The current challenges associated with fecal microbiota transplantation (FMT) include, but are not limited to, the need for standardization and regulation, the identification of suitable donors, and the risk of transferring pathogenic bacteria to patients, particularly those who are immunocompromised. Additionally, FMT may be significantly more expensive than standard antibiotics. While there may be some detriments to FMT, the benefits include a restored immune system and repairing host damage by enriching it with bacteria conducive to gut stabilization [55]. Furthermore, there have been studies that have reported that patients who had ulcerative colitis (UC) achieved remission and an increase in SCFAs such as butyrate and butyrate-producing agents six months after receiving FMT [34,56]. On the other hand, challenges unique to probiotic supplementation include potential interactions with other medications, determining the efficacy and proper dosing, and the risk of contamination with pathogenic bacteria, which may cause more harm than benefit to the patient.

### 5.3. Ethical Considerations

Research into the microbiome, particularly in vulnerable populations such as pregnant women and infants, raises a range of ethical considerations. Informed consent, privacy protection, and the possible lasting impacts of microbiome interventions are among the key issues. Tackling these concerns is of the utmost importance to uphold ethical standards in research and guarantee fair access to emerging therapies.

## Figures and Tables

**Table 2:** Microbiome-targeted interventions for allergy prevention and treatment.

Intervention Type	Specific Agents/Examples	Mechanism of Action	Target Population	Clinical/Experimental Outcomes	Evidence Type
Probiotics	*Lactobacillus rhamnosus GG*; *Bifidobacterium breve*	↓ IgE; ↑ IgA; SCFA production	Infants, children, adults	Reduced allergic responses	Clinical + preclinical
Prebiotics	Dietary fibers, oligosaccharides	Promote beneficial bacteria; ↑ SCFAs	All ages	Improved gut health	Clinical (variable)
Synbiotics	Probiotic + prebiotic combinations	Synergistic microbiome modulation	Infants, children	Enhanced immune tolerance	Emerging
Postbiotics	SCFAs (butyrate, acetate)	Treg induction; anti-inflammatory	All ages	Reduced inflammation	Preclinical + early clinical
FMT	Donor microbiota transfer	Restore microbial diversity	Adults	Reduced allergy (animal models)	Preclinical + Phase I
Dietary	Mediterranean, high-fiber diets	↑ SCFAs; ↓ inflammation	Adolescents, adults	Improved metabolic profile	Observational/interventional
Engineered microbiota	Genetically modified bacteria	Targeted immune modulation	Experimental	Precision therapy potential	Experimental
Bacteriophage therapy	Phage-based targeting	Selective bacterial modulation	Experimental	Correct dysbiosis	Emerging
Environmental	Indoor microbiome control	Alter exposure diversity	Adolescents, adults	Reduced rhinitis risk	Observational

## Data Availability

No new data were created or analyzed in this study. Data sharing is not applicable to this article.
